# Strong-correlation induced high-mobility electrons in Dirac semimetal of perovskite oxide

**DOI:** 10.1038/s41467-018-08149-y

**Published:** 2019-01-21

**Authors:** J. Fujioka, R. Yamada, M. Kawamura, S. Sakai, M. Hirayama, R. Arita, T. Okawa, D. Hashizume, M. Hoshino, Y. Tokura

**Affiliations:** 1 Department of Applied Physics, University of Tokyo, Tokyo, 113-8656 Japan; 2 PRESTO, Japan Science and Technology Agency, Kawaguchi, 332-0012 Japan; 3 RIKEN Center for Emergent Matter Science (CEMS), Wako, 351-0198 Japan; 4 Present Address: Graduate School of Pure and Applied Science, University of Tsukuba, Tsukuba, Ibaraki Japan

## Abstract

Electrons in conventional metals become less mobile under the influence of electron correlation. Contrary to this empirical knowledge, we report here that electrons with the highest mobility ever found in known bulk oxide semiconductors emerge in the strong-correlation regime of the Dirac semimetal of perovskite CaIrO_3_. The transport measurements reveal that the high mobility exceeding 60,000 cm^2^V^−1^s^−1^ originates from the proximity of the Fermi energy to the Dirac node (ΔE < 10 meV). The calculation based on the density functional theory and the dynamical mean field theory reveals that the energy difference becomes smaller as the system approaches the Mott transition, highlighting a crucial role of correlation effects cooperating with the spin-orbit coupling. The correlation-induced self-tuning of Dirac node enables the quantum limit at a modest magnetic field with a giant magnetoresistance, thus providing an ideal platform to study the novel phenomena of correlated Dirac electron.

## Abstract (web summary)

Electron correlation normally makes electrons less mobile, but it is still not clear when correlation becomes very strong in Dirac semimetals. Here, Fujioka et al. report a very high electron mobility exceeding 60,000 cm^2^V^−1^s^−1^ in correlated Dirac semimetal of perovskite CaIrO3, due to the enhanced electron correlation nearby the Mott transition.

## Introduction

The topological (Dirac/Weyl) semimetal, a class of materials with the low-energy electronic excitations described by Dirac/Weyl electron of relativistic theory, exemplifies an active frontier of modern condensed matter science. Triggered by the research on three-dimensional Dirac/Weyl semimetals, various topological semimetals such as the nodal-line semimetal have been successively identified, and the scope of Dirac/Weyl electron in solid is expanding even beyond the scheme of relativistic particle in high-energy physics^1,2^. A distinguished feature of Dirac/Weyl electron with massless or small effective mass character is its extremely high mobility, yielding a variety of unusual quantum transports. The representative examples are the room temperature quantum Hall effect in graphene^3^ or chiral anomaly in the Weyl semimetal^4^. Beyond the single-particle physics, currently, there is a growing interest in the strong-correlation effect on Dirac/Weyl electrons. In the magnetic quantum limit, wherein all electrons occupy the lowest Landau level (LL), the effect of Coulomb interaction is crucial, which offers a novel opportunity to study collective phenomena of relativistic electrons. Indeed, the bulk fractional quantum Hall effect, valley ordering, or nematic quantum liquid have been argued for bithmuth^5–7^. Recent research proposes the ordering of Weyl electron at extremely high magnetic fields above 80 T in the Weyl semimetal TaAs^8^.

Another route to realize the strong-correlation effect of the highly mobile Dirac/Weyl electrons is to utilize correlated electron materials. So far, only a few candidates of correlated Dirac/Weyl semimetals have been experimentally identified. Among them, the 5*d*-transition-metal oxide is a promising class of materials, where the interplay between electron correlation and relativistic spin–orbit interaction yields the Dirac/Weyl semimetal coupled to the Mott physics or magnetism^9,10^. Indeed, the first prediction of magnetic Weyl semimetal has been done for the pyrochlore-type iridates, wherein signatures of correlated Weyl electron have been identified as anomalous magneto-transport phenomena^9–12^. However, the strong electron correlation usually decreases the electron mobility along the promotion of electron localization, and thus quantum transport of relativistic electron would be hardly observed in this class of materials.

Contrary to this naive expectation, however, we report here for the strongly correlated Dirac semimetal CaIrO_3_ that the combination of electron correlation and spin–orbit coupling cooperatively yields highly mobile electrons with the mobility exceeding 60,000 cm^2^ V^−1^ s^−1^, the largest among the oxide semiconductors, as well as the unique quantum oscillation with giant positive magnetoresistivity ratio of 5500%.

## Results

### Material

**Fig. 1:**
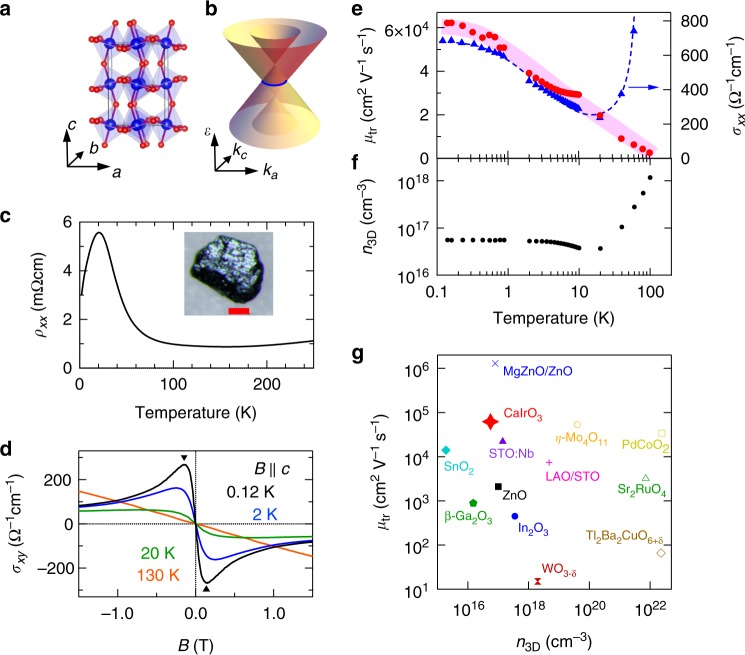
Unusually large transport mobility in perovskite CaIrO_3_. **a** The crystal structure and **b** the sketch of Dirac-like dispersion near the line node (blue line). The primitive vectors *a*, *b*, and *c* are defined in the orthorhombic notation with space group *Pbnm*. **c** Temperature dependence of the resistivity. The inset is the photograph of single-crystalline CaIrO_3_. The red bar denotes the length of 100μm. **d** The Hall conductivity *σ*_*xy*_ vs. *B* (*B* = *μ*_0_*H*, with *μ*_0_ the vacuum permittivity) at various temperatures. The reciprocal field of peak or dip as indicated by triangles corresponds to the averaged transport mobility *μ*_tr_. **e**, **f** show the *μ*_tr_ and density (*n*_3D_) of highest mobility carrier as a function of temperature. The temperature dependence of electrical conductivity *σ*_*xx*_ is also shown. **g** The *μ*_tr_ of typical high-mobility oxide semiconductors and metals^21, 32–40^ as well as the heterostructure of (Mg,Zn)O/ZnO^41^ and LAO(LaAlO_3_)/STO(SrTiO_3_)^42^. The carrier densities of heterostructures are derived by assuming the effective channel thickness of 10 nm

Perovskite *A*IrO_3_ (*A* = Ca, Sr) crystallizes in an orthorhombic perovskite structure with the GdFeO_3_-type lattice distortion as shown Fig. 1a. The electronic state nearby the Fermi energy (*E*_F_) is mainly composed of nearly half-filled *j*_eff_ = 1/2 multiplet of 5*d* orbitals of Ir^4+^ ion, leading to a semimetallic state with a few electron and hole pockets^13–16^^,^. The first principles calculation argues that this material is a nodal-line semimetal protected by the nonsymmorphic crystalline symmetry, wherein the conduction band and valence band cross along a closed line in momentum space as depicted in Fig. 1b^13^. For SrIrO_3_ in the thin film form, the electron pocket with Dirac-like dispersion has been identified by the angle-resolved photoemission spectroscopy, and the Dirac node positions below *E*_F_ by more than 50 meV^17^. A striking feature of this material is the electron correlation can be effectively controlled by the one-electron bandwidth. Indeed, the modest reduction of one-electron bandwidth by the dimensional control or chemical substitution in SrIrO_3_ causes the semimetal-to-antiferromagnetic insulator transition^18–20^. The one-electron bandwidth can also be controlled by the GdFeO_3_-type lattice distortion (or Ir-O-Ir bond angle distortion) via replacing Sr with Ca; the effective electron correlation is enhanced approximately by 20% in CaIrO_3_ compared with SrIrO_3_, as measured from the increase of the bond angle distortion in the perovskite^21^. In this context, CaIrO_3_ would be located in even closer proximity to the Mott transition, offering a rare opportunity to study the Mott criticality of Dirac electron. Nevertheless, the hallmark of Dirac electron such as the highly mobile electron showing the quantum transport has not been detected. One major obstacle is the difficulty in preparing the perovskite crystal structure of CaIrO_3_, which is a metastable form at ambient pressure. To overcome this issue, we have employed a high-pressure synthesis technique and succeeded in synthesizing high-quality single crystals of perovskite CaIrO_3_.

### Transport measurements

Figure 1c shows the temperature dependence of resistivity of CaIrO_3_. The resistivity shows a metallic temperature dependence above 150 K, whereas a notable peak emerges at around 20 K. The sign of Hall conductivity *σ*_*xy*_ indicates that the dominant carrier is electron type (Fig. 1d). At 0.12 K, a dispersion-type profile with a peak and dip is observed around ±0.14 T, but diminishes as temperature increases. By employing the semiclassical Boltzmann formula (Supplementary Note 1), we extracted the density (*n*_3D_) and mobility (*μ*_tr_) of electrons. As shown in Fig. 1e, the mobility is enhanced with decreasing temperature and reaches 62,000 cm^2^ V^−1^ s^−1^ at 0.12 K, which is exceptionally high among the typical bulk oxide semiconductors (see Fig. 1g). The enhancement of mobility is in parallel to that of electrical conductivity, while the carrier density is nearly temperature independent below 20 K (Fig. 1f). Therefore, it is likely that the peak of resistivity can be understood from the counter-balance of carrier density and mobility, and the highly mobile electrons govern the transport at low temperatures.

**Fig. 2:**
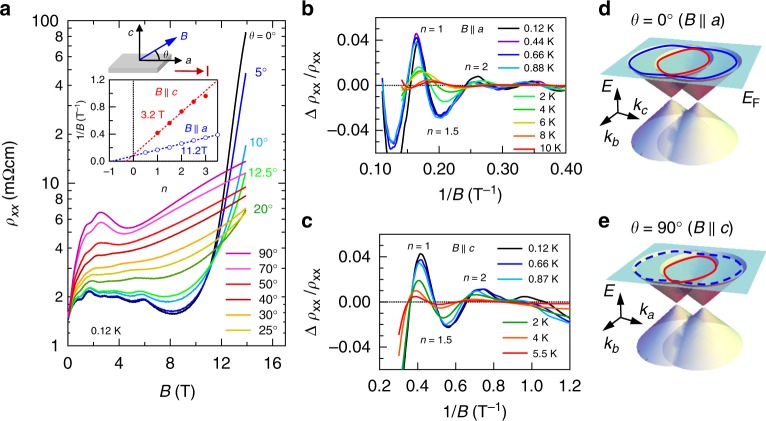
Shubnikov–de-Haas (SdH) oscillations and giant positive magnetoresistivity. **a** The angular dependence of resistivity *ρ*_*xx*_ at 0.12 K. The SdH oscillation for 2–9 T is clear for *B*||*a*, while that for 1–3 T is clear even for *B*||*c*. The giant positive magnetoresistivity is observed above 9 T nearby *B*||*a*. Inset shows the Landau index plot of the oscillation for *B*||*a* and *B*||*c* and illustration of measurement geometry for *B* and electrical current *I* (||*a*). The tilting angle *θ* is defined as zero for *B*||*a*. **b**, **c** Oscillatory component at various temperatures for *B*||*a* and for *B*||*c*. **d**, **e** Illustration of band dispersion nearby the line node described in *k*_*b*_–*k*_*c*_ plane and *k*_*a*_–*k*_*b*_ plane. The red and blue line denotes the cross-section of inner- and outer-Fermi surface (FS), respectively. The SdH oscillation of outer-FS is not visible for *B*||*c*, which is indicated by dashed lines

Figure 2a shows the magnetoresistivity at 0.12 K measured in varying magnetic field (*B*) and its direction within the *ac* plane. For *B*||*a* (*θ* = 0°), the resistivity initially increases up to 2 T, decreases during 2–9 T, and exhibits two orders of magnitude increase above 9 T. By tilting the magnetic field from the *a*-axis, the giant magnetoresistivity above 9 T is suppressed and the resistivity moderately increases up to 14 T. Moreover, clear Shubnikov–de-Haas (SdH) oscillations are observed below 10 K. For *B*||*a*, two SdH oscillations are observed during 1–3 and 2–9 T. The latter (high-field oscillation) diminishes as *θ* increases, while the former (low-field oscillation) becomes clear at *θ* = 90° (*B*||*c*). Figure 2b, c shows the oscillation components after subtraction of the non-oscillating background for high-field oscillation (*B*||*a*) and low-field oscillation (*B*||*c*), respectively. We plot the LL fan diagram to extract the oscillation frequency *B*_F_ and phase shift *φ*, following the Lifshitz–Onsager quantization rule *B*_F_/*B* = *n* − *φ* (inset to Fig. 2a). Here, the peak and valley positions, which are consistent with the second derivative of *ρ*_*xx*_ ( = d*ρ*_*xx*_^2^/d*B*^2^), are assigned to integer and half-integer, respectively. The linearity of the fan plot up to the quantum limit may be a consequence of small Zeeman splitting. First, we analyzed the high-field oscillation for *B*||*a*. The extracted *B*_F_ and *φ* are 11.2T and −0.9, respectively. The corresponding extremal area of Fermi surface (FS) is determined to be *S*_F_ = 1.0 × 10^−3^ Å^−2^ and the carrier density estimated from *S*_F_ is 2.1 × 10^17^ cm^−3^. Because of the absence of oscillation above 9 T and the small extremal area of FS (*B*_F_ = 11.2 T), it is likely that 9 T is sufficient to reach the quantum limit, where electrons of this FS start to occupy the lowest LL. We extracted the cyclotron mass *m*_c_, Fermi velocity *v*_F_, and Dingle temperature *T*_D_ according to the Lifshitz–Kosevich formula,

1 $$\Delta \rho _{xx}{\mathrm{/}}\rho _{xx} \propto \exp \left( { - x_{\mathrm{D}}} \right)\frac{x}{{\sinh \left( x \right)}}\cos 2{\mathrm{\pi }}\left( {B_{\mathrm{F}}{\mathrm{/}}{B}{\mathrm{ + }}\varphi } \right),$$

with *x*_D_ = 2*π*^2^*k*_B_*T*_D_/*ħω*_c_ and *x* = 2*π*^2^*k*_B_*T*/*ħω*_c_. The estimated *m*_c_, *v*_F_, and *T*_D_ are 0.31 ± 0.04*m*_0_, with *m*_0_ the free electron mass, 6.9 ± 0.6 × 10^4^ m s^−1^, and 3.5 K, respectively (Supplementary Fig. 3a). We similarly analyzed the low-field oscillation for *B*||*c*. The oscillation frequency is extracted to be *B*_F_ = 3.2 T corresponding to the *S*_F_ = 3.0 × 10^−4^ Å^−2^, which is nearly one-third of that for high-field oscillation. Moreover, *m*_c_, *v*_F_, *T*_D_, and *φ* are determined to be 0.12 ± 0.04*m*_0_, 8.7 ± 1.2 × 10^4^ m s^−1^, 4.5 K, and −0.3, respectively. The extracted parameters for both oscillations are summarized in Supplementary Table 2.

### Calculations based on density functional theory and dynamical mean field theory

**Fig. 3:**
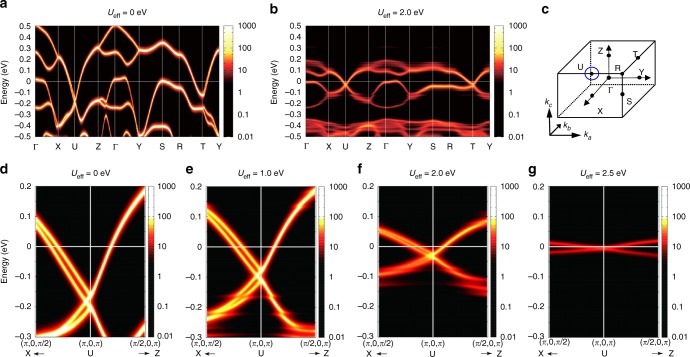
Band structure calculated by density functional theory and dynamical mean field theory. **a**, **b** Overview of band structure with the Hubbard *U*_eff_ = 0 and 2.0 eV. The scale bar denotes the magnitude of spectral function. **c** Illustration of momentum space and position of Dirac line node (blue line). **d**–**g** The magnified view of band structure around the Dirac line node with different *U*_eff_. The Dirac line node approaches the Fermi energy with increasing *U*_eff_. The Dirac-like dispersion is significantly renormalized for *U*_eff_ = 2.5 eV, which is a precursory phenomenon of the Mott criticality. The averaged Fermi velocity is 2.0 × 10^5^, 1.8 × 10^5^, 8.1 × 10^4^, and 2.0 × 10^4^m s^−1^ at *U*_eff_ = 0, 1.0, 2.0, and 2.5 eV, respectively

To understand the observed transport properties, we carried out the calculation using the density functional theory combined with the dynamical mean field theory (DMFT) to calculate a realistic electronic structure subject to the Coulomb interaction *U*_eff_ and relativistic spin–orbit interaction (see Methods). The band structure with *U*_eff_ = 0 eV is displayed in Fig. 3a. The bands lying from −0.4 to 0.5 eV dominantly consist of *j*_eff_ = 1/2 state. Highly dispersing bands along X-U-Z line and R-T-Y line are crossing *E*_F_, yielding electron pockets. Specifically, the Dirac-like linearly dispersing bands are seen nearby the U-point (*k*_*a*_ = 0, *k*_*b*_ = *π*, *k*_*c*_ = *π*); the band crossing constitutes a closed loop encircling the U-point on the *k*_*b*_ = *π* plane (Fig. 3c) at −0.2 eV. Interestingly, the two bands are dispersing in nearly parallel in a wide energy regime, yielding two FS neighboring each other. On the other hand, the bands along R-T-Y, Z-Γ-Y, and Y-S-R lines show the parabolic dispersion. Figure 3b shows the band structure with *U*_eff_ = 2.0 eV. The bandwidth of *j*_eff_ = 1/2 state is renormalized into the energy regime from −0.2 to 0.2 eV while keeping the presence of line node. Notably, both the electron and hole pockets shrink compared with the case of *U*_eff_ = 0 eV with keeping the charge neutrality. Indeed, bands around Y-S-R line are pushed down and the hole pocket around S-point vanishes. Figure 3d–g shows the magnified view nearby the U-point. With increasing *U*_eff_, the bandwidth is significantly renormalized and *v*_F_ is reduced from 2.0 × 10^5^ m s^−1^ at *U*_eff_ = 0 eV to 8.0 × 10^4^ m s^−1^ at *U*_eff_ = 2.0 eV (Supplementary Figure 8). Consequently, the energy of the line node relative to *E*_F_ is squeezed from −0.2 eV at *U*_eff_ = 0 to −0.03 eV at *U*_eff_ = 2.0 eV. This large renormalization is a precursory phenomenon of the Mott transition. The systematic evolution of band structure upon increasing *U*_eff_ suggests the remarkable scenario that the Fermi energy is most closely tuned to the Dirac node at the Mott criticality.

### Fermi surface

Among the electron pockets around U-point and those around T-point, we conclude that the formers are the plausible candidates of the highly mobile electron by following reasons. First, the observed Fermi velocity is consistent with the calculated band structure. Second, the angular dependence of *S*_F_ is consistent with that expected from the electron pockets around U-point (Supplementary Note 3). In this context, it is conceivable that the low (high)-field oscillation is ascribed to the inner-FS (outer-FS) inherent to the Dirac-like dispersion as illustrated in Fig. 2d, e. Assuming that the Fermi velocities of inner- and outer-bands are equivalent and that their anisotropy can be neglected, the diameter of line node is estimated to be about 0.008 Å^−1^, which is as small as about 1/70 of the reciprocal lattice units *π*/*a* (Supplementary Note 3). Moreover, the energy of line node is estimated to be 5–8 meV below *E*_F_. Such a nearly perfect tuning of *E*_F_ to the line node and the good agreement of *v*_F_ between the theory (*U*_eff_ = 2.0 eV) and experiment verify the aforementioned scenario.

### Giant magnetoresistivity

**Fig. 4:**
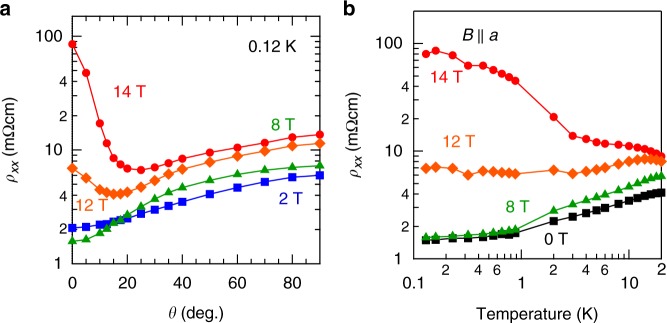
Angular and temperature dependence of giant magnetoresistivity. **a** The resistivity at various magnetic fields vs. *θ* at 0.12 K. **b** Temperature dependence of resistivity for *B*||*a*

Having established these results, we discuss the origin of giant magnetoresistivity observed above 9 T. Figure 4a shows the *θ*-dependence of resistivity at various magnetic fields. As *θ* increases, the resistivity at 14 T rapidly decreases; the resistivity at *θ* = 20° is <1/10 of that at *θ* = 0°. On the contrary, the resistivity at lower fields moderately increases as a function of *θ*. Figure 4b shows the temperature dependence of resistivity at *θ* = 0° (*B*||*a*). With decreasing temperature, the resistivity increases at 14 T, but shows a metallic behavior below 8 T. The close similarity between the angular and temperature dependence of giant magnetoresistivity and those of high-frequency oscillation (Supplementary Figure 3 and Figure 5) suggests that the highly resistive state is inherent to the quantum limit of the outer-FS. Under sufficiently high magnetic fields in the quantum limit, the magnetic length $l_{B} = 1{\mathrm{/}}\sqrt {eB}$ is reduced to the scale of Fermi wavelength, leading to the quasi-one-dimensional (1D) state dispersing along the field direction. The important consequence of quasi-1D confinement of Dirac electron is the gap opening or electron localization due to the strong enhancement of electron correlation or disorder effect as well as the inter-node mixing effect^22–25^. Indeed, the temperature dependence of resistivity at *B*(||*a*)=14 T can be described by the variable range hopping model with electron correlation or localized Tomonaga–Luttinger liquid model (Supplementary Figure 9). Thus, it is likely that the giant magnetoresistivity is ascribed to the gap-out of line node or the electron localization promoted by the magnetically induced quasi-1D confinement, which can be realized at the modest field as enabled by the nearly perfectly tuned line node. Such a quantum transport in the quantum limit has been rarely realized in existing Dirac/Weyl semimetals, which may characterize the emergent topological property of this correlated Dirac semimetal.

## Discussions

We demonstrated that the electron correlation cooperative with the relativistic spin–orbit coupling yields unusually highly mobile electrons exceeding 60,000cm^2^ V^−1^ s^−1^ in the Dirac semimetal perovskite CaIrO_3_. From the analysis of SdH oscillation, the Fermi energy is nearly pinned at the Dirac line node (within ~8 meV) and the magnetic quantum limit is reached at the modest magnetic field of about 9 T. In the quantum limit, we identified a field-direction-sensitive giant positive magnetoresistivity with a ratio of about 5500% at 0.12 K and 14 T, suggesting the mass gap opening or the electron localization promoted by the quasi-1D confinement. The manifestation of highly mobile Dirac electrons in strongly correlated materials opens a new stage of research on quantum phenomena in topological materials.

## Methods

### Sample preparation and characterization

Single crystals of perovskite CaIrO_3_ were grown by solid-state reaction using the cubic-anvil-type facility. The materials were heated up to 1200 °C under 1 GPa, remained there for 10 min, and then quenched to room temperature. The high-pressure synthesis can provide the high-quality samples with the right stoichiometry (i.e., half band-filling). This is crucial to pin the Fermi energy nearby the Dirac line node. The typical size of crystal is about 0.5 × 0.5 × 0.3 mm^3^ as shown in the inset to Fig. 1b. The crystal orientation was determined using the X-ray diffractometer.

The crystallographic symmetry is orthorhombic *Pbnm*, in agreement with the previous report according to powder X-ray diffraction^26^. We analyzed the crystal structure on the basis of the collected data sets of single-crystal X-ray diffraction. Supplementary Figure 1 shows the typical X-ray diffraction pattern taken at 300 K. In total, the 14,436 reflections (799 independent reflections) up to sin *θ*/*λ* = 0.92 Å^−1^ were observed. No superlattice reflections which violate the *Pbnm* symmetry are discernible within the experimental accuracy. The derived structural parameters are listed in Supplementary Table 1.

### Measurement of resistivity and Hall resistivity

Measurements of resistivity and Hall resistivity were performed by standard four-terminal geometry with indium electrode. The measurements above 2 K were done by using the Physical Property Measurement System (Quantum Design). The measurement below 1 K was performed by using the dilution refrigerator equipped with the 14 T superconducting magnet (Oxford instruments). We employed a standard lock-in technique with a fixed excitation current (1–10μA) at low frequency (1–10 Hz).

### Electronic structure calculation

We used the Perdew–Burke–Ernzerhof exchange-correlation functional^27^ as implemented in the WIEN2K program^28^. The muffin tin radii (*R*_MT_) of 2.11, 2.00, and 1.77 bohr were used for Ca, Ir, and O, respectively. The maximum modulus for the reciprocal vectors *K*_max_ was chosen such that *R*_MT_ *K*_max_ = 7.0 and a 10 × 10 × 10 **k**-mesh in the first Brillouin zone was used. We then constructed the Wannier functions for the *t*_2*g*_ bands, using the WIEN2WANNIER^29^ and the WANNIER90^30^ codes.

With these Wannier functions, we constructed a tight-binding model with three *t*_2*g*_ Wannier orbitals at each of four equivalent Ir sites in the unit cell. We considered the Hubbard-type onsite Coulomb interaction *U*_eff_ on the basis diagonalizing the local Hamiltonian, where *U*_eff_ is an orbital independent density–density-type interaction.

The correlation effects due to *U*_eff_ were taken in within the DMFT^31^, which counts the full local correlations while neglecting the nonlocal correlations, which are expected to be small in the present three-dimensional material. The impurity problem in the DMFT was solved with an exact diagonalization method, where the DMF is expressed by a finite number of bath sites. We used nine bath sites for this. Applying the Lanczos method to the finite-size Hamiltonian, we calculated the ground state and the self-energy. For real-frequency axis, we introduced an energy-smearing factor of 5 meV. This self-energy on real-frequency axis was used to calculate the spectral function plotted in Fig. 3. The calculated results show that the system persists to be metallic up to a relatively large value of *U*_eff_. This is presumably because the semimetallic density of states of this material will not have a much benefit of the energy reduction due to the opening of the Mott gap.

## Supplementary information

Supplementary Information

Peer Review File

Description of Additional Supplementary Files

Supplementary Data 1

Supplementary Data 2

## Data Availability

All data needed to evaluate the conclusions in the paper are present in the paper and/or the supplementary materials. Additional data requests should be addressed to the corresponding authors.

## References

[1] Armitage NP, Mele EJ, Vishwanath A (2018). Weyl and Dirac semimetals in three dimensional solids. Rev. Mod. Phys..

[2] Bradlyn B (2016). Beyond Dirac and Weyl fermions: unconventional quasiparticles in conventional crystals. Science.

[3] Novoselov KS (2007). Room-temperature quantum Hall effect in graphene. Science.

[4] Xiong J (2015). Evidence for the chiral anomaly in the Dirac semimetal Na_3_Bi. Science.

[5] Behnia K, Balicas L, Kopelvich Y (2007). Signatures of electron fractionalization in ultraquantum bismuth. Science.

[6] Li L (2008). Phase transitions of dirac electrons in bismuth. Science.

[7] Feldman BE (2016). Observation of a nematic quantum Hall liquid on the surface of bismuth. Science.

[8] Ramshaw BJ (2018). Quantum limit transport and destruction of the Weyl nodes in TaAs. Nat. Commun..

[9] Wan X, Turner AM, Vishwanath A, Savrasov SY (2011). Topological semimetal and Fermi-arc surface states in the electronic structure of pyrochlore iridates. Phys. Rev. B.

[10] Witczak-Krempa W, Kim YB (2012). Topological and magnetic phases of interacting electrons in the pyrochlore iridates. Phys. Rev. B..

[11] Ueda K (2018). Spontaneous Hall effect in the Weyl semimetal candidate of all-in all-out pyrochlore iridate. Nat. Commun..

[12] Juyal A, Agarwal A, Mukhopadhyay S (2018). Negative longitudinal magnetoresistance in the density wave phase of Y_2_Ir_2_O_7_. Phys. Rev. Lett..

[13] Carter JM (2012). Semimetal and topological insulator in perovskite iridates. Phys. Rev. B..

[14] Zhang H, Haule K, Vanderbilt D (2013). Effective *J* = 1/2 insulating state in Ruddlesden–Popper iridates: an LDA+DMFT study. Phys. Rev. Lett..

[15] Zhao JG (2008). High-pressure synthesis of orthorhombic SrIrO_3_ perovskite and its positive magnetoresistance. J. Appl. Phys..

[16] Fujioka J, Okawa T, Yamamoto A, Tokura Y (2017). Correlated Dirac semimetallic state with unusual positive magnetoresistance in strain-free perovskite SrIrO_3_. Phys. Rev. B..

[17] Nie Y (2015). Interplay of spin–orbit interactions, dimensionality, and octahedral rotations in semimetallic SrIrO_3_. Phys. Rev. Lett..

[18] Matsuno J (2015). Engineering a spin-orbital magnetic insulator by tailoring superlattices. Phys. Rev. Lett..

[19] Cui Q (2016). Slater insulator in iridate perovskites with strong spin–orbit coupling. Phys. Rev. Lett..

[20] Imada M, Fujimori A, Tokura Y (1998). Metal–insulator transitions. Rev. Mod. Phys..

[21] Tufte ON, Chapman PW (1967). Electron mobility in semiconducting strontium titanate. Phys. Rev..

[22] Yang KY, Lu YM, Ran Y (2011). Quantum Hall effects in a Weyl semimetal: possible application in pyrochlore iridates. Phys. Rev. B.

[23] Wei H, Chao SP, Aji V (2012). Excitonic phases from Weyl semimetals. Phys. Rev. Lett..

[24] Zhang XX, Nagaosa N (2017). Tomonaga–Luttinger liquid and localization in Weyl semimetals. Phys. Rev. B.

[25] Kim P, Ryoo JH, Park CH (2017). Breakdown of the chiral anomaly in Weyl semimetals in a strong magnetic field. Phys. Rev. Lett..

[26] Cheng JG (2011). High-pressure synthesis and physical properties of perovskite and post-perovskite Ca_1−*x*_Sr_*x*_IrO_3_. Phys. Rev. B.

[27] Perdew JP, Burke K, Ernzerhof M (1996). Generalized gradient approximation made simple. Phys. Rev. Lett..

[28] Blaha, P. et al. http://www.wien2k.at.

[29] Kunes J (2010). Wien2wannier: from linearized augmented plane waves to maximally localized Wannier functions. Comput. Phys. Commun..

[30] Mostofi AA (2008). wannier90: a tool for obtaining maximally-localised Wannier functions. Comput. Phys. Commun..

[31] Georges A, Kotliar G, Krauth W, Rozenberg MJ (1996). Dynamical mean-field theory of strongly correlated fermion systems and the limit of infinite dimensions. Rev. Mod. Phys..

[32] Fonstad CG, Rediker RH (1971). Electrical properties of high‐quality stannic oxide crystals. J. Appl. Phys..

[33] Look DC (1998). Electrical properties of bulk ZnO. Solid State Commun..

[34] Weiher RL (1962). Electrical properties of single crystals of indium oxide. J. Appl. Phys..

[35] Oishi T, Koga Y, Harada K, Kasu M (2015). High-mobility β-Ga_2_O_3_($/bar{2}01$) single crystals grown by edge-defined film-fed growth method and their Schottky barrier diodes with Ni contact. Appl. Phys. Exp..

[36] Berak JM, Sienko MJ (1970). Effect of oxygen-deficiency on electrical transport roperties of tungsten trioxide crystals. J. Solid State Chem..

[37] Hicks CW (2012). Quantum oscillations and high carrier mobility in the delafossite PdCoO_2_. Phys. Rev. Lett..

[38] Mackenzie AP, Maeno Y (2003). The superconductivity of Sr_2_RuO_4_ and the physics of spin-triplet pairing. Rev. Mod. Phys..

[39] Vignolle B (2008). Quantum oscillations in an overdoped high-Tc superconductor. Nature.

[40] Inoue M, Ohara S, Horisaka S, Koyano M, Negishi H (1988). Phys. Stat. Sol. (b).

[41] Falson J (2016). MgZnO/ZnO heterostructures with electron mobility exceeding 1 × 10^6^ cm^2^/Vs. Sci. Rep..

[42] Xie Y (2014). Quantum longitudinal and Hall transport at the LaAlO_3_/SrTiO_3_ interface at low electron densities. Solid State Commun..

