# Supplementary material for: Strong-correlation induced high-mobility electrons in Dirac semimetal of perovskite oxide
Source: Nat Commun. 2019 Jan 21;10:362. doi: 10.1038/s41467-018-08149-y (PMC6341165; doi:10.1038/s41467-018-08149-y)
Supplement: Supplementary file 1 — Supplementary Information [file 41467_2018_8149_MOESM1_ESM.pdf]

Supplementary information for "Strong-correlation induced ultrahigh mobility electrons in Dirac semimetal of perovskite oxide"

J. Fujioka[1,2*†], R. Yamada[1], M. Kawamura[3], S. Sakai[3], M. Hirayama[3], R. Arita[1,3], T. Okawa[1], D. Hashizume[3], M. Hoshino[2,3] and Y. Tokura[1,3*].

Correspondence to: fujioka@ims.tsukuba.ac.jp; tokura@riken.jp

J. Fujioka *et al*

Supplementary Note 1: Analysis of resistivity and Hall resistivity

Supplementary Figure 2a shows the temperature dependence of resistivity for various samples. All samples show similar temperature dependence; the resistivity moderately decreases as temperature decreases from 300 K to 100 K and shows a peak around 20 K. We determined the carrier density and mobility from the Hall conductivity in the scheme of semiclassical Boltzmann theory,

$$\sigma_{xy}=\sum_{i=1}^{2} n_i e\mu_{tr,i}\frac{\mu_{tr,i}B}{1+(\mu_{tr,i}B)^2} \quad (1)$$

Here, n_i and $\mu_{tr,i}$ are the carrier density and transport mobility of i-th carrier. The best fitting curve is obtained with assuming two kinds of carriers. The fitting curves for the S9 sample at 0.12 K are exemplified in Supplementary Fig. 2b with $n_1=5.5\times10^{16}$ cm^{-3}, $\mu_{tr1}=6.2\times10^4$ cm^{2}V^{-1}s^{-1}, $n_2=2.0\times10^{17}$ cm^{-3} and $\mu_{tr2}=2.1\times10^3$ cm^{2}V^{-1}s^{-1}, respectively. The signs of fitted curves are negative for $B > 0$, indicating that the two kinds of carriers are electron-type. The parameters of highest mobility carrier do not significantly depend on the fitting procedure of another; the lower mobility carrier constitutes a broad peak lying in the high field regime as demonstrated by the green curve in Supplementary Fig. 2b and gives a least effect in the low field regime where the high mobility carrier governs the Hall conductivity. The μ_{tr} of the highest mobility carrier is plotted as a function of temperature in Supplementary Fig. 2c. As discussed in the main text, μ_{tr} increases as temperature decreases and reaches 62,000 cm^{2}V^{-1}s^{-1} for S9 at 0.12 K.

Supplementary Note 2: Analysis of Shubnikov de Haas oscillation

For the analysis of SdH oscillations, we adopted the formula of resistivity[1,2] expressed as

$$\frac{\Delta\rho_{xx}}{\rho_{xx}}=\frac{5}{2}\sum_{r=1}^{\infty} b_r\cos2\pi[(B_F/B+\gamma)r-\delta]+R \quad (2),$$

$$b_r=\sqrt{\frac{\hbar\omega_c}{2E_F r}}\exp(-x_D r)\frac{xr}{\sinh(xr)}\cos\left(\frac{\pi m_c g r}{2m_0}\right) \quad (3),$$

$$R=\frac{3\hbar\omega_c}{8E_F}\left[\sum_{r=1}^{\infty} b_r\{\alpha_r\cos2\pi(B_F/B+\gamma)r+\beta_r\sin2\pi(B_F/B+\gamma)r\}-\ln(1-e^{-2x_D})\right] \quad (4),$$

$$\alpha_r=2\sum_{s=1}^{\infty}\sqrt{\frac{r}{s(r+1)}}e^{-2x_D s} \quad (5)$$

$$\beta_r=\sum_{s=1}^{r-1}\sqrt{\frac{r}{s(r-s)}} \quad (6)$$

Given that the g-factor is about 2[3], the energy scale of Zeeman splitting is much smaller than cyclotron energy in the investigated magnetic field regime. For simplicity, we have neglected the last factor of Supplementary Eq. (3) [cos ($\pi r m_c g/2m_0$)]. We have also neglected the terms of higher harmonics ($r > 1$) and R, taking into account the magnitude of m_c, B_F and T_D. Then, Supplementary Eq. (2) is simplified to Eq. (1) in the main text. Supplementary Fig. 3a demonstrates the thermal damping of oscillation amplitude and fits by Eq. (1) of the main text for the high-field oscillation for $B\|a$. The extracted m_c slightly depends on Landau index n and is determined to be $0.31\pm0.04m_0$ with m_0 the free electron mass. The oscillation amplitude at n=1 cannot be fitted by a single value of m_c; the amplitude shows moderate temperature dependence above 3 K, but is steeply enhanced below 3 K, resulting in $m_c=0.34m_0$ at lower temperatures and $m_c=0.13m_0$ at higher temperatures, respectively. The Dingle temperatures are determined by the

Dingle plot of ln[$\Delta\rho_{xx}/\rho_{xx}$ Bsinh(x)] vs 1/B as shown in Supplementary Fig. 3b. The Dingle plot at 0.12 K yields T_D=3.5 K, corresponding to the quantum life time τ_Q=3.5×10^{-13} s.

We have performed similar analysis for the low-field oscillation of $B||c$ (Supplementary Figs. 3d and 3e). The temperature dependence of oscillation amplitude at n=1 again cannot be reproduced by a single value of m_c as in the case of high-field oscillation (Supplementary Fig. 3d). The deviation from Lifshitz-Kosevich formula at n=1 suggests that the terms of higher harmonics ($r > 1$) in Supplementary Eq. (2) or breakdown of the rigid band scheme, which may be a precursory phenomenon of gap opening or electron localization inherent to the quantum limit, cannot be neglected. The carrier densities estimated by assuming an isotropic Fermi surface are qualitatively consistent with the results of Hall conductivity. In addition, we also performed the fitting of $\Delta\rho_{xx}/\rho_{xx}$ with Eq. (1) of the main text. As shown in Supplementary Figs. 3c and 3f, Eq. (1) of the main text adequately reproduces the high-field oscillation for $B \parallel a$, but poorly does the low-field oscillation for $B \parallel c$.

Supplementary Note 3: Assignment of Fermi surface and estimation of size of line node

One of the major difference between the electron pockets around U-point ($k_a = 0$, $k_b = \pi$, $k_c = \pi$) and those around T-point ($k_a = \pi$, $k_b = 0$, $k_c = \pi$) is in the anisotropy of extremal cross-section of Fermi surface S_F. To clarify the anisotropy, we investigated the SdH oscillation under the tilted magnetic field. Supplementary Figures 5a and 5b show the high- and low-field SdH oscillations ($\Delta\rho_{xx}/\rho_{xx}$) when the magnetic field is tilted within the ac-plane at 0.12 K. The peak and dip of the high-field oscillation move to larger $1/B$ as the tilting angle θ increases, while those of the low-field oscillation show more moderate θ-dependence. Supplementary Figure 5c shows the peak and dip of oscillatory component as a function of $1/B$ and θ. The low-field oscillation (open symbols) is discernible in a wide angular regime $0 \leq \theta \leq 90°$, whereas the high-field oscillation (filled symbols) is not clear for $\theta > 40°$.

First, we focus on the θ-dependence of the low-field oscillation. Supplementary Figure 5d shows the θ-dependence of S_F. The S_F moderately decreases with decreasing θ from 90° ($B \parallel c$). We also plot the expected θ-dependence of S_F which is simulated with the calculated band structure. Here, the absolute value of simulated S_F is set as a free parameter, since E_F has not been exactly reproduced by the *ab-initio* calculation at the precision required for the present purpose. The observed angular dependence of S_F is consistent with the electron pockets around U-point rather than those around T-point.

Next, we consider the θ-dependence of the high-field oscillation. The S_F and m_c of the high-field oscillation are nearly constant for $\theta \leq 10°$, but suddenly decrease and appear to merge into those of the low-field oscillation for $\theta \geq 13°$. This sudden change cannot be reproduced by the FS predicted by the calculated band structure, given that the FS or magnetic (cyclotron) orbit is not significantly reconstructed by the magnetic field. One possible scenario is the magnetic breakdown orbit, which is often seen in metals with multiple Fermi surface; the magnetic orbit changes via the quantum tunneling of electron between the two neighboring FSs, accompanying the change in the S_F and m_c. Indeed, the steep angular dependence of S_F or m_c has been ascribed to the angle sensitive magnetic breakdown in the metal with multiple Fermi surfaces[4]. Because the line node can be gapped out under the magnetic field tilted from high symmetry axis in the present material[5], one plausible scenario is that the field induced band reconstruction smears out or pushes up the SdH oscillation of outer-FS to much higher field regime, and consequently the diminishing quantum orbit of outer-FS partially overlaps with that of the inner-FS via the

quantum tunneling for 13° ≤ θ ≤ 40°. Indeed, the modest magnetic field of about 5 T may be sufficient to induce tunneling among them with the momentum separation of the inner- and outer-FSs (about 0.008 Å^{-1}).

We also explored the SdH oscillation under the magnetic field tilted within the *bc*-plane (ω). Supplementary Figures 6a and 6b show the high-field and low-field SdH oscillations, respectively, measured at 2 K. Here, the measurement geometry is shown in Supplementary Fig. 6c. The high-field oscillation is discernible for ω ≤ 15°, whereas the low-field one is observed in wide range of 45° ≤ ω ≤ 90° (=$B \parallel c$). Supplementary Figure 6d shows the Landau index plot of both oscillations. The S_F of high-field oscillation for $B \parallel b$ is comparable with that for $B \parallel a$. The angular dependence of S_F is plotted in Supplementary Fig. 6e. The S_F of low-field oscillation increases with decreasing ω. This behavior is consistent with the electron pocket nearby U-point (solid curve in Supplementary Fig. 6e). On the other hand, the S_F of the high-field oscillation suddenly changes around ω = 10°, as in the case for $B \parallel ac$-plane. We anticipate that the magnetic breakdown between the inner- and outer-Fermi surfaces of Dirac-like dispersion occurs even for $B \parallel bc$-plane.

Having identified the plausible electron pockets, next we discuss E_F in the Dirac like dispersion. There are two possibilities whether E_F is located above or below the "band crossing point at U-point" as shown in Supplementary Figs. 7b and 7d. The geometrical shape of Fermi surface(s) is two concentric spheroids in the former case, but is a torus in the latter case (Supplementary Figs. 7c and 7e). Although both cases yield two SdH oscillations with different frequency, we conclude that the former case is more plausible for the following reasons; (a) both kinds of carriers extracted from σ_{xy} are electron-type, (b) the angular dependence of S_F of the inner-FS is moderate in a wide angle range both within *ac*-plane and within *bc*-plane, and (c) the S_F of the outer FS for $B \parallel a$ is comparable with that for $B \parallel b$.

By assuming that the linearly dispersing bands are parallel to each other and the anisotropy of FS can be neglected, the diameter of the line node can be estimated by the difference between the k_F of the inner- and outer-FSs as illustrated in Supplementary Figs. 7a and 7b. The "averaged" k_F of the outer-FS is determined to be about 0.018 Å^{-1} from the SdH oscillation for $B \parallel b$. Since the k_F of the inner-FS could not be accurately extracted for $B \parallel b$, we employed the k_F determined for $B \parallel c$ (=0.01 Å^{-1}). In this way, the diameter of the nodal loop is approximately determined to be 0.008 Å^{-1}.

Finally, we remark the phase shift of SdH oscillation. A distinguished feature of the line node is that electrons acquire a nontrivial π Berry phase around the loop encircling the line node, but do a trivial phase around a loop parallel to the plane of the line node[6]. It is well-known that the Berry phase manifests itself as the phase shift of the SdH oscillation. According to Ref.6, the outer FS with electron character should give rise to the phase shift φ = -5/8 when the magnetic field is parallel to or perpendicular to the nodal loop. The phase shift of the inner FS is expected to be 5/8 or 1/8 in the case of torus type Fermi surface with a low Fermi energy. Within the experimental accuracy, the observed value of the high-field oscillation (φ= - 0.9) suggests the outer-FS of line node, which also supports our interpretation.

Supplementary Note 4: Resistivity in the quantum limit

To quantify the insulator-like behavior at high magnetic field, we analyzed the temperature dependence of electrical conductivity σ_{xx} (= $1/\rho_{xx}$) at 14 T by thermal activation model, variable range hopping model and the model of Tomonaga-Luttinger liquid with disorder. The resistivity

above 1 K can be fitted by the Arrhenius model $\sigma_{xx} = \sigma_0 + A_0\exp(T_0/T)$, yielding the thermal activation energy T_0 = 1.9 ±0.6 K (Supplementary Fig. 9a). Here the σ_0 and A_0 are the temperature independent conductivity stemming from residual carriers and a constant, respectively. Since T_0 is too small to be ascribed to the field induced mass gap, it would correspond to the thermal activation energy of nearly localized carriers due to the spatially varying potential landscape as observed in the two dimensional electron gas[7,8].

In this context, we have examined the variable range hopping and model of Tomonaga-Luttinger liquid with disorder[9]. Supplementary Figures 9b-d display the fitting by the variable range hopping (VRH) model, the model of Tomonaga-Luttinger liquid with disorder and the VRH model with Coulomb interaction, respectively. The corresponding temperature dependence of resistivity is expressed as Supplementary Eqs. 7-9, respectively.

$$\sigma_{xx} = \sigma_0 + A_1\exp(T_1/T)^{1/(1+d)} \qquad (7)$$

$$\sigma_{xx} = \sigma_0 + B_1 T^{\alpha} \qquad (8)$$

$$\sigma_{xx} = \sigma_0 + \frac{A_2}{\sqrt{T}}\exp(T_2/T)^{1/(1+d)} \qquad (9)$$

Here, T_1, T_2, A_1, A_2, B_1 and α are temperature independent fitting parameters. The VRH model and the model of Tomonaga-Luttinger liquid with disorder are consistent with the observed resistivity below 1 K. On the other hand, the VRH model with Coulomb interaction appears to be appropriate in a wider temperature range. Although we cannot uniquely determine the best model, it is likely that the thermally assisted hopping transport governs the low temperature transport.

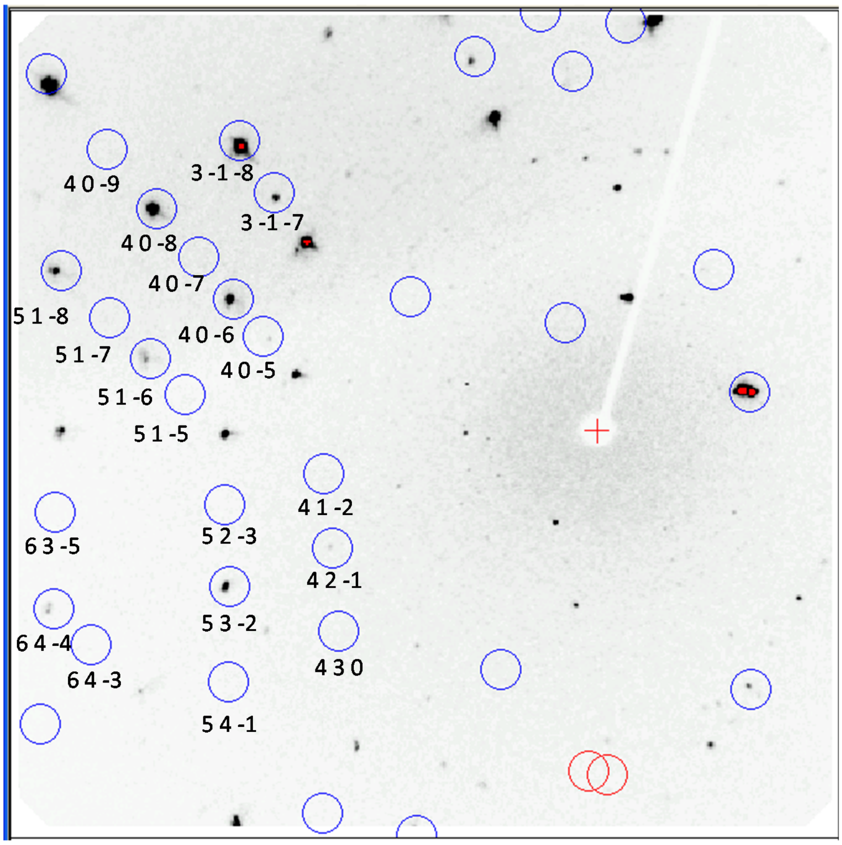

Fig. 1. X-ray diffraction pattern of single crystalline $CaIrO_3$.
The number is the Bragg reflection index.

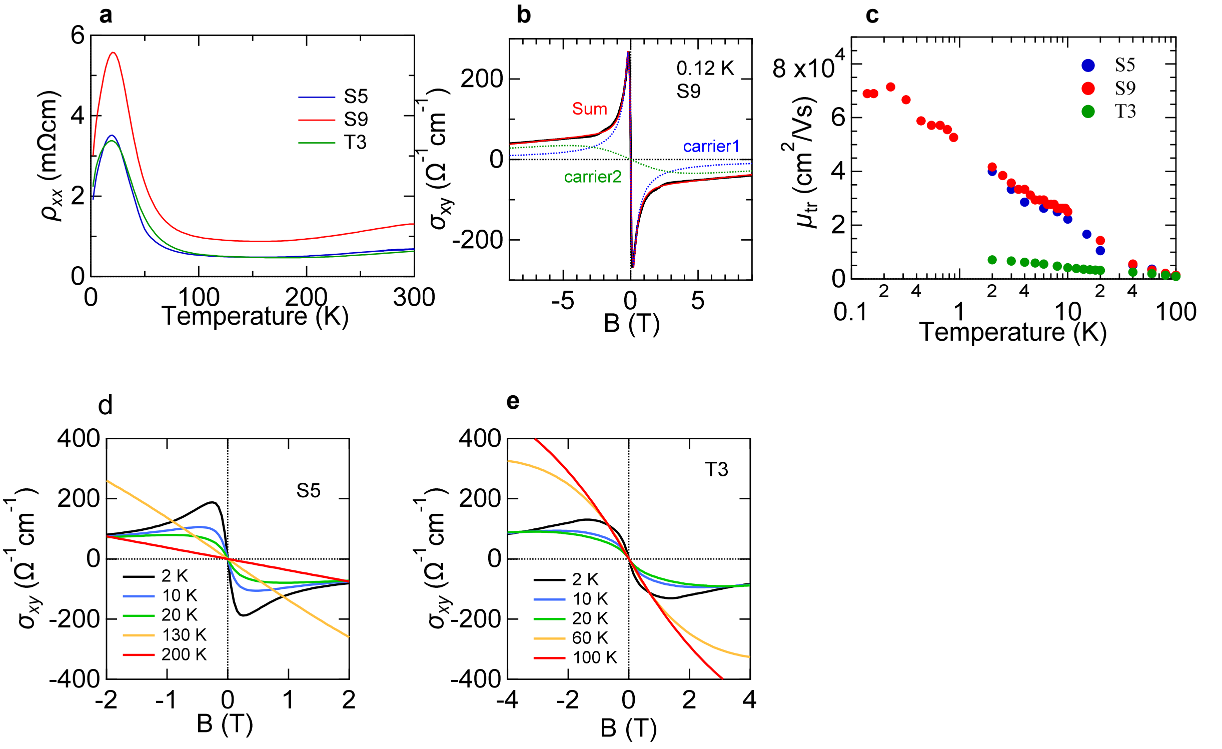

Fig. 2. Comparison of resistivity and Hall conductivity in various samples.

a Temperature dependence of resistivity for samples S5, S9 and T3. **b** The experimental data and fitting curve within the semiclassical Boltzmann transport scheme for sample S9, where two kinds of carriers (1 and 2) are assumed. **c** The transport mobility versus temperature. **d** and **e** Hall conductivity versus *B* in sample S5 and in sample T3, respectively.

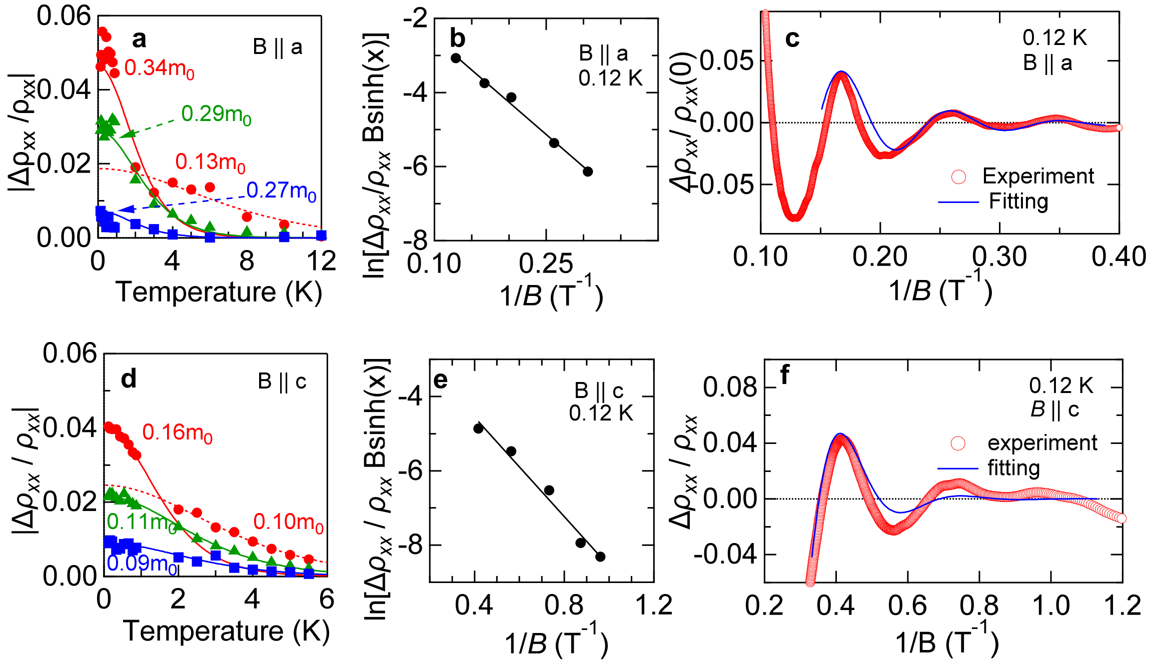

Fig. 3. Temperature and magnetic field dependence of SdH oscillation.

a&d Temperature dependence of the oscillation amplitude at various Landau indices (circle: n=1, triangle: n=1.5, square: n=2) for high- and low-field oscillations and a fitting with Eq. (1) of the main text. The effective mass is extracted at each Landau index. **b&e** The Dingle plots of log[$d\rho_{xx}/\rho_{xx} \cdot B\sinh(x)$] versus $1/B$ at 0.12 K for the high- and low-field oscillations. **c&f** Experimental data and a fit by the Lifshitz-Kosevich formula potted against $1/B$ for the high- and low-field oscillations.

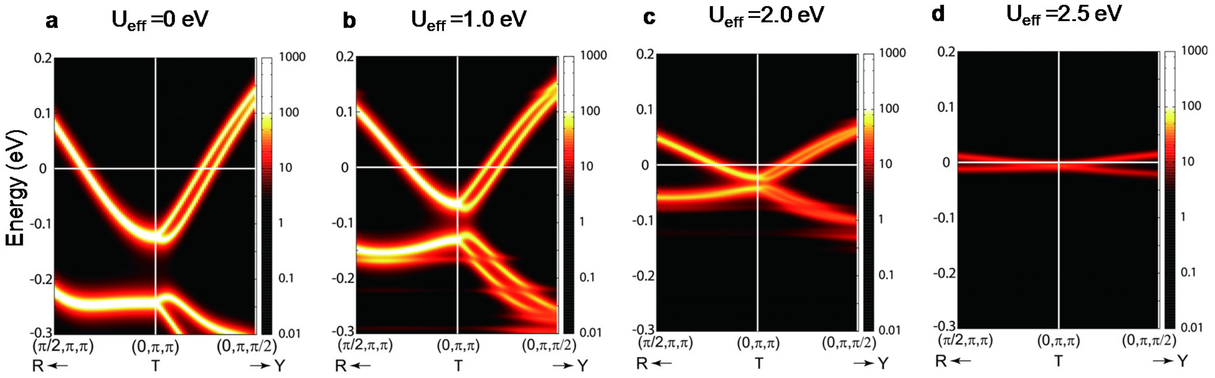

Fig. 4. Band structure around T-point.

a-d The band structure around T-point for $U_{\mathrm{eff}} = 0$, 1.0, 2.0 and 2.5 eV. The color denotes the magnitude of spectral function.

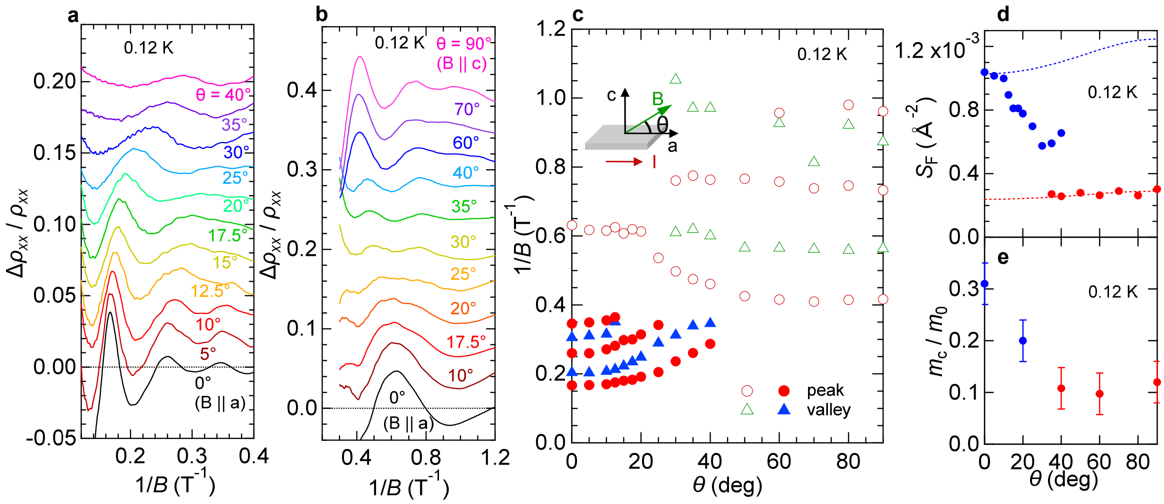

Fig. 5. Angular dependence of SdH oscillation for magnetic field tilted in *ac*-plane.

a&b The high- and low-field SdH oscillations at 0.12 K. **c** The SdH oscillation as a function of θ and $1/B$. Open (closed) circle and triangle denote the peak and dip of low (high) field oscillation, respectively. The low-field oscillation is observed for $1/B > 0.4$ T^{-1} in the regime $0° < \theta < 90°$. On the other hand, the high-field oscillation is observed for $1/B < 0.4$ T^{-1} in the regime $0° < \theta < 40°$. Inset shows the illustration of measurement geometry about the magnetic field and electrical current. **d&e** The extremal cross-section of Fermi surface S_F and effective mass of electron m_c/m_0 plotted as a function of θ, respectively. Blue (red) circle denotes S_F and m_c/m_0 corresponding to the high-field (low-field) oscillation. The dashed line is the S_F nearby the U-point stemming from the Dirac-like band dispersion, which is simulated with the calculated band structure. The sudden deviation of the high-field oscillation may be attributed to the magnetic breakdown. The SdH oscillation stemming from the electron pockets around T-point is not visible. The uncertainty of effective mass is derived from the statistical deviation associated with the analysis of SdH oscillation.

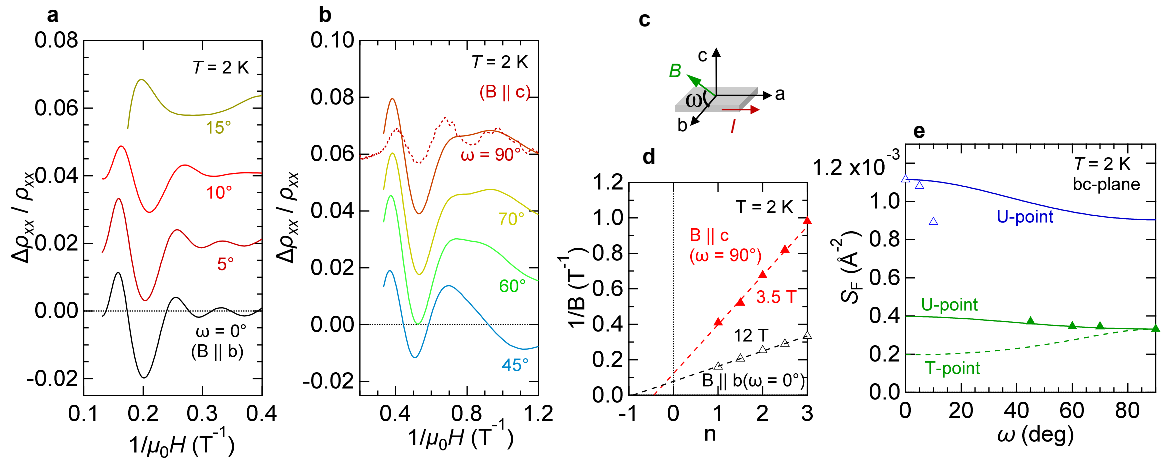

Fig. 6. Angular dependence of SdH oscillation for magnetic field tilted in *bc*-plane.

a&b The high- and low-field SdH oscillations at 2 K. The dashed line in **b** denotes the second derivative of ρ_{xx} ($-d\rho^2_{xx}/dB^2$) at ω=90°. **c** The illustration of geometry of magnetic field and electrical current. **d** Landau index plot of high-field oscillation for $B \parallel b$ and low-field oscillation for $B \parallel c$. **e** The extremal cross-sectional area of Fermi surface S_F versus tilted angle ω in the *bc*-plane. The solid and dashed curves denote the ω dependence of S_F for electron pockets around U-point and T-point, respectively, which are simulated with the calculated band structure. The electron pockets around U-point are qualitatively consistent with the experimental results. The rapid reduction of S_F of high-field oscillation above 10° may be ascribed to the magnetic breakdown between the inner- and outer-FSs.

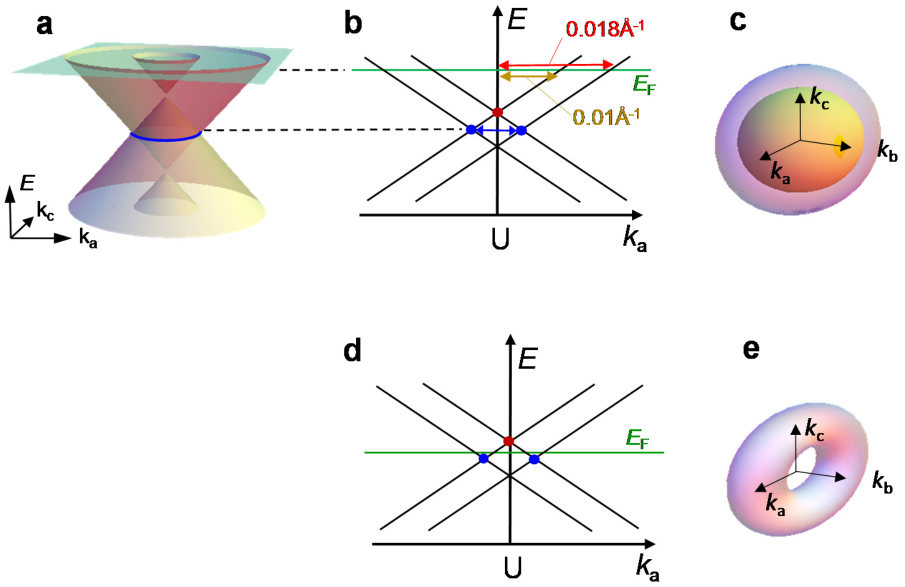

Fig. 7. Illustration of band dispersion nearby the Dirac line node.

a&b The illustration of band dispersion within the k_a-k_c plane and its cross-section along the k_a-line. The line node is marked by the blue line or blue points. The green plane or line denote the Fermi energy. **c** The Fermi surface when the Fermi energy is above the band crossing at U-point (brown circle). **d&e** The illustration of band dispersion along k_a-line with the Fermi energy close to the line node and the corresponding Fermi surface.

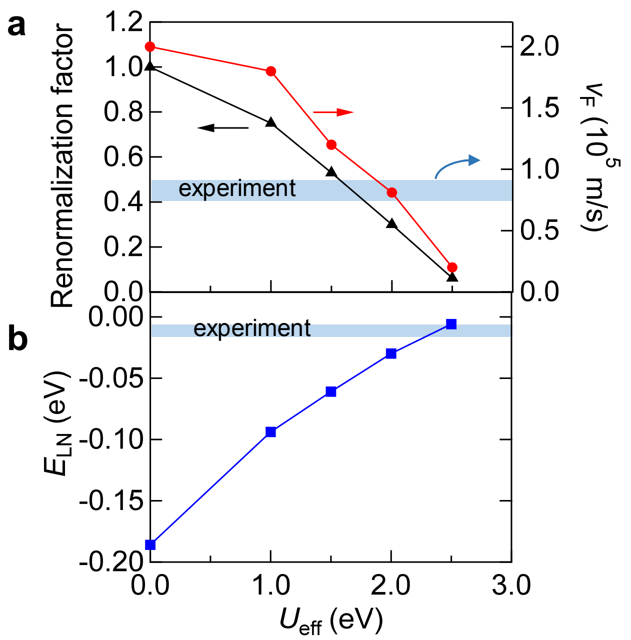

Fig. 8. The U_{eff} dependence of band parameters.

a The renormalization factor and Fermi velocity v_F plotted as a function of U_{eff}. The v_F was evaluated for the Fermi surface around the U point, in the direction along the U-X line. The v_F along the U-Z [U-R] line is slightly (by ~10%) larger [smaller] than the plotted value. The hatched bar denotes v_F determined by the experiment (the SdH oscillations). **b** The energy of the nodal line E_{LN}, measured from the Fermi energy, is determined by the peak energy of the spectral function along the U-X line. The horizontal line denotes E_{LN} evaluated from the experiment (the SdH oscillations). With increasing U_{eff}, v_F decreases monotonically along with the renormalization factor, whereas E_{LN} asymptotically approaches E_F as the precursory phenomena of the Mott criticality. On the basis of the magnitude and systematic variation of v_F and E_F, the results of U_{eff}=2.0 eV may be most consistent with the experimental results.

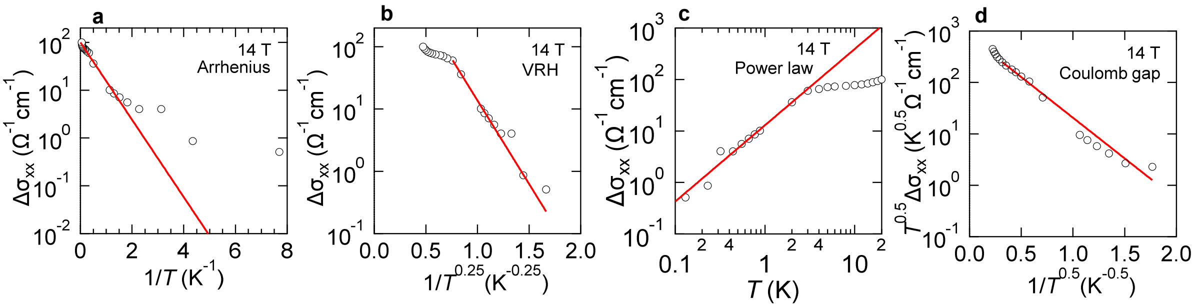

Fig. 9. Temperature dependence of electrical conductivity at 14 T.

a-c Temperature dependence of electrical conductivity at 14T plotted as a function of $1/T$, $1/T^{0.25}$, T for the Arrhenius, variable range hopping (VRH) and Tomonaga-Luttinger model with disorder[35] **d** The value of $T^{0.5}\sigma_{xx}$ versus $T^{0.5}$ for the Coulomb gap model.

Atom	site	x	y	z	U_{11}	U_{22}	U_{33}	U_{12}	U_{13}	U_{23}
Ir	4*a*	0	0	0	0.0146(3)	0.0180(3)	0.0075(3)	-0.00002(3)	-0.00002(2)	-0.00005(3)
Ca	4*c*	0.48318(12)	0.0604(2)	1/4	0.0182(3)	0.0215(5)	0.0122(3)	0	0	-0.0009(2)
O1	4*c*	0.6036(5)	0.4650(5)	1/4	0.0193(10)	0.0250(11)	0.0088(8)	0	0	-0.0002(9)
O2	8*d*	0.1933(3)	0.3011(4)	0.0522(2)	0.0184(7)	0.0218(7)	0.0133(6)	-0.0012(5)	0.0019(5)	-0.0028(6)

Table 1. Crystallographic data for perovskite $CaIrO_3$ at 300 K.

Space group *Pbnm* (No. 62), lattice parameter a= 5.3597(5) Å, b= 5.6131(4) Å, c = 7.6824(8) Å, Z=4, V= 231.12(4) Å^3 The reliability factors are R=0.0540, R_W=0.1440, GOF(Goodness of fit) = 1.147. In the table, x, y and z are the fractional coordinates. Anisotropic atomic displacement parameters are represented as U_{11}, U_{22}, U_{33}, U_{12}, U_{13} and U_{23} in units of (Å^2). The bond angles of Ir-O1-Ir and Ir-O2-Ir are 145.90(16) ° and 146.76(10)°, respectively.

Table 2. Parameters extracted from the SdH oscillations.

Geometry	S_F[Å^{-2}]	k_F[Å^{-1}]	n_{3D} (cm^{-3})	m_c/m_0	v_F(ms^{-1})	T_D (K)	τ_Q (s)
Low-freq. (B||c)	3.0×10^{-4}	0.010	3.2×10^{16}	0.12 ± 0.04	$8.7\pm1.2\times10^{4}$	4.5	2.7×10^{-13}
High-freq. (B||a)	1.0×10^{-3}	0.018	2.1×10^{17}	0.31 ± 0.04	$6.9\pm0.6\times10^{4}$	3.5	3.5×10^{-13}

References.

(1) G. Landwehr, E. I. Rashba, in Landau Level Spectroscopy:Modern Problems in Condensed Matter Sciences (North-Holland, Amsterdam, 1991), vol. 27.2.
(2) H. Murakawa, *et al*., Detection of Berry's Phase in a Bulk Rashba Semiconductor. Science **342**, 1490-1493 (2013).
(3) S. Bahr, *et al*., Low-energy magnetic excitations in the spin-orbital Mott insulator Sr_2IrO_4. Phys. Rev. B **89**, 180401(R) (2014).
(4) S. Pezzini *et al*., Unconventional mass enhancement around the Dirac nodal loop in ZrSiS. Nature Phys. **14**, 178-183 (2018).
(5) Y. Chen, Y-.M. Lu and H-.Y. Kee, Topological crystalline metal in orthorhombic perovskite iridates. Nature Commun. **6**, 6593 (2015).
(6) C. Li, *et al*., Rules for Phase Shifts of Quantum Oscillations in Topological Nodal-Line Semimetals. Phys. Rev. Lett. **120**, 146602 (2018).
(7) A. J. Bestwick, E. J. Fox, X. Kou, L Pan, K. L.Wang, and D. Goldhaber-Gordon, Precise Quantization of the Anomalous Hall Effect near Zero Magnetic Field. Phys. Rev. Lett. **114**, 187201 (2015).
(8) M. Kawamura, R. Yoshimi, A. Tsukazaki, K. S. Takahashi, M. Kawasaki, and Y. Tokura, Current-Driven Instability of the Quantum Anomalous Hall Effect in Ferromagnetic Topological Insulators. Phys. Rev. Lett. **119**, 016803 (2017).
(9) X-X Zhang and N. Nagaosa, Tomonaga-Luttinger liquid and localization in Weyl semimetals. Phys. Rev. B **95**, 205143 (2017).
