# Supplementary material for: Strong-correlation induced high-mobility electrons in Dirac semimetal of perovskite oxide
Source: Nat Commun. 2019 Jan 21;10:362. doi: 10.1038/s41467-018-08149-y (PMC6341165; doi:10.1038/s41467-018-08149-y)
Supplement: Supplementary file 3 — Description of Additional Supplementary Files [file 41467_2018_8149_MOESM3_ESM.pdf]

Description of Additional Supplementary Files

Supplementary Data 1

The source file of Supplementary Fig. 5e with statistical values.

Supplementary Data 2

The structural parameters of perovskite $CaIrO_3$.
