# Supplementary material for: Strong-correlation induced high-mobility electrons in Dirac semimetal of perovskite oxide
Source: Nat Commun. 2019 Jan 21;10:362. doi: 10.1038/s41467-018-08149-y (PMC6341165; doi:10.1038/s41467-018-08149-y)
Supplement: Supplementary file 2 — Peer Review File [file 41467_2018_8149_MOESM2_ESM.pdf]

Editorial Note: This manuscript has been previously reviewed at another journal that is not operating a transparent peer review scheme. This document only contains reviewer comments and rebuttal letters for versions considered at Nature Communications.
